# Descriptive Epidemiology of New York City Older Adult Patients With Multiple Chronic Conditions

**DOI:** 10.5888/pcd21.230444

**Published:** 2024-08-01

**Authors:** Sarah Conderino, John A. Dodson, Yuchen Meng, Mark G. Weiner, Catherine Rabin, Wilson Jacobs, Parampreet Bakshi, Melissa Lee, Jenny Uguru, Lorna E. Thorpe

**Affiliations:** 1 Department of Population Health, New York University Grossman School of Medicine, New York, New York; 2 Department of Population Health Sciences, Weill Cornell Medicine, New York, New York; 3 New York City Health and Hospitals, New York, New York

## Abstract

We characterized comorbidity profiles and cardiometabolic risk factors among older adults with multiple chronic conditions (MCCs) in New York City using an intersectionality approach. Electronic health record data were obtained from the INSIGHT Clinical Research Network on 367,901 New York City residents aged 50 years or older with MCCs. Comorbidity profiles were heterogeneous. The most common profile across sex and racial and ethnic groups was co-occurring hypertension and hyperlipidemia; prevalence of these 2 conditions differed across groups (4.7%–7.3% co-occurrence alone, 65.1%–88.0% with other conditions). Significant sex and racial and ethnic differences were observed, which may reflect accumulated disparities in risk factors and health care access across the life course.

**Summary**

**What is already known on this topic?**

Co-occurrence of multiple chronic conditions (MCCs) is a significant public health concern among older adults, with differential burden and profiles by demographic subgroups.

**What is added by this report?**

We descriptively characterized older adults with MCCs using a large, diverse patient sample from New York City. We identified significant potential disparities in comorbidity burden and cardiovascular risk factors by the intersection of race and ethnicity and sex.

**What are the implications for public health practice?**

Public health interventions are needed to prevent chronic disease onset, and community–clinical interventions are needed to improve disease management among minority populations and enhance quality of life.

## Objective

Co-occurrence of multiple chronic conditions (MCCs) is a significant public health concern among older adults in the US, affecting an estimated 64% of those aged 65 years or older and contributing to worse health outcomes or quality of life (1). Prior work has demonstrated that the burden of MCCs differs by demographic factors (1,2). However, few studies have characterized MCCs using an intersectional approach to understand variation of comorbidity burden by sex and race and ethnicity, which may reflect, among other factors, effects of interdependent systems of discrimination or oppression, such as sexism and racism, on health outcomes (3). Large patient samples afforded by electronic health record (EHR) data offer the opportunity to examine people with MCCs at the granularity required for such an approach (4,5).

The goal of this study was to capitalize on the large sample size and demographic diversity found in an EHR-based data system in New York City (NYC) to characterize the burden and cardiometabolic risk factors of older adult patients with MCCs. We hypothesized that these would meaningfully differ by race and ethnicity and sex. More broadly, assessing the descriptive epidemiology of patients with MCCs by the intersection of race and ethnicity and sex may facilitate a deeper understanding of underlying structural factors influencing chronic disease onset.

## Methods

This cross-sectional study was conducted through use of data from the INSIGHT Clinical Research Network (CRN). INSIGHT is the largest urban CRN in the US, capturing data of more than 17 million diverse patients from 7 academic health care systems in NYC and Houston, Texas (6). This study was approved by the NYU Langone institutional review board.

To avoid the effects of pandemic-associated death or diagnosis misclassification due to disruption in care, our study population was restricted to NYC residents aged 50 years or older with at least 1 ambulatory visit in the 6 months before COVID-19 pandemic onset (September 7, 2019–March 6, 2020) and 2 or more chronic conditions (https://github.com/condes01/covid-shutdown). Those with a diagnosis for Alzheimer disease, dementia, or metastatic cancer were excluded. To examine intersectionality by major sex and racial and ethnic groups, we stratified individuals into 8 categories: female Asian, male Asian, female Black, male Black, female Latino, male Latino, female White, and male White.

Primary outcomes included prevalence of each chronic condition, defined by having 1 or more *International Classification of Diseases* *10th Revision* (ICD-10) diagnosis codes for the condition from January 1, 2016, through March 6, 2020 (https://github.com/condes01/covid-shutdown). Secondary outcomes were cardiometabolic risk factors of systolic blood pressure (SBP), cholesterol, hemoglobin A1c (HbA1c), and weight, obtained from vital signs or laboratory measurements taken during ambulatory visits in 2019. We calculated individuals’ average risk factor value, excluding biologically implausible measurements (https://github.com/condes01/covid-shutdown). We then defined binary variables to indicate elevated cardiometabolic risk factors of obesity (body mass index ≥30 kg/m^2^); high BP (SBP ≥140 mm Hg); high cholesterol (low-density lipoprotein [LDL] cholesterol ≥160 mg/dL, high-density lipoprotein cholesterol <40 mg/dL for men or <50 mg/dL for women, total cholesterol ≥240 mg/dL, or triglycerides ≥200 mg/dL); and elevated HbA1c (≥6.5%).

We calculated prevalence and 95% CIs of each chronic condition and characterized comorbidity profiles overall and by major sex and racial and ethnic group. As a sensitivity analysis, we assessed age-adjusted differences in prevalence by using logistic regression models, controlling for 5-year age group. We explored differences in cardiometabolic risk factors by group using analysis of variance tests for continuous outcomes and χ^2^ tests for categorical outcomes.

## Results

In total, 367,901 older adults had MCCs in the INSIGHT study population. A higher proportion of these patients were female (59%) than male (41%), and most patients were White (38%) and from medium poverty neighborhoods (43%). A substantial proportion of patients had unknown race and ethnicity (20%).

Substantial heterogeneity of chronic condition combinations existed in this population (Figure). The most prevalent conditions were hypertension (79.3%) and hyperlipidemia (72.1%), and the most common comorbidity profile was the co-occurrence of these 2 conditions (6% alone, 59% in combination with other conditions), overall and across all sex and race and ethnicity groups, although the prevalence of these conditions differed across groups. Prevalence of hypertension was highest among Black women (87.6%) and Black men (88.0%), and hyperlipidemia was highest among Asian (78.9%) and White men (79.0%) (Table 1).

**Figure:**
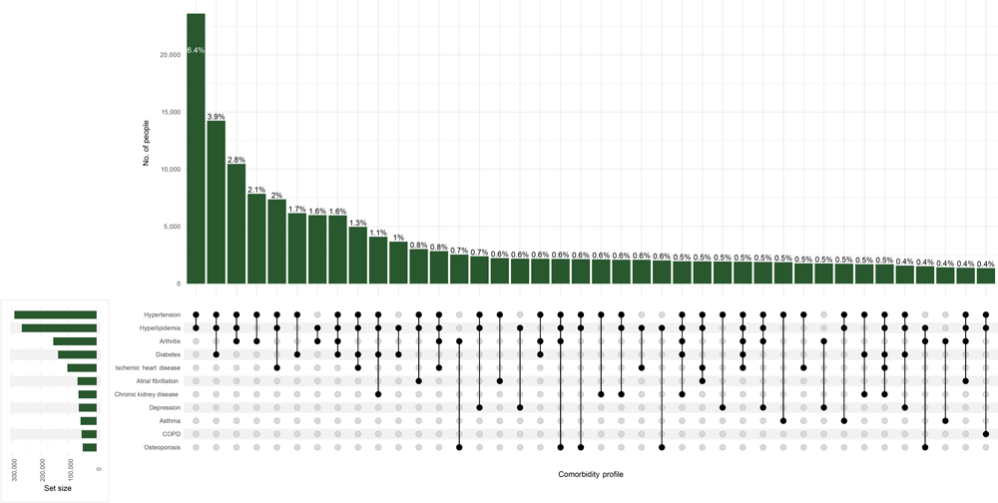
Leading comorbidity profiles among New York City residents aged 50 years or older with multiple chronic conditions receiving care at NYC INSIGHT facilities, September 2019–March 2020.

**Table 1:** Demographic Characteristics and Comorbidity Prevalence Among New York City Residents Aged 50 Years or Older with Multiple Chronic Conditions Receiving Care at NYC INSIGHT Facilities, September 2019 Through March 2020

Demographic characteristic	Asian (n = 12,684)	Black (n = 53,403)	Latino (n = 75,431)	White (n = 138,552)
**Female sex, no.**	**7,048**	**35,527**	**48,645**	**75,866**
**Age, no. (%), y**				
50–64	2,771 (39.32)	15,372 (43.27)	20,288 (41.71)	18,543 (24.44)
≥65	4,277 (60.68)	20,155 (56.73)	28,357 (58.29)	57,323 (75.56)
**Poverty level, no. (%)^a^**				
Low	1,823 (25.87)	4,056 (11.42)	3,243 (6.67)	30,056 (39.62)
Medium	3,546 (50.31)	15,632 (44.00)	17,194 (35.35)	35,865 (47.27)
High	1,331 (18.88)	9,046 (25.46)	15,074 (30.99)	8,225 (10.84)
Very high	348 (4.94)	6,792 (19.12)	13,130 (26.99)	1,708 (2.25)
Unknown	0 (0)	1 (0)	4 (0.01)	12 (0.02)
**Comorbidities, % (95% CI)**				
AMI	3.1 (2.7–3.6)	2.7 (2.6–2.9)	2.9 (2.8–3.1)	3.4 (3.3–3.5)
Arthritis	41.4 (40.3–42.6)	50.5 (50.0–51.0)	49.8 (49.3–50.2)	50.4 (50.0–50.7)
Asthma	12.8 (12.0–13.6)	20.9 (20.5–21.3)	26.7 (26.3–27.1)	13.4 (13.2–13.6)
Atrial fibrillation	14.4 (13.6–15.2)	12.6 (12.2–12.9)	12.1 (11.8–12.4)	20.9 (20.6–21.2)
Breast cancer	10.0 (9.3–10.7)	8.0 (7.7–8.3)	7.3 (7.0–7.5)	12.6 (12.4–12.9)
Chronic kidney disease	13.9 (13.1–14.8)	22.9 (22.4–23.3)	17.0 (16.6–17.3)	9.0 (8.8–9.2)
Colorectal cancer	1.3 (1.1–1.6)	1.5 (1.4–1.6)	1.4 (1.3–1.5)	1.7 (1.6–1.8)
COPD	9.8 (9.1–10.5)	14.0 (13.6–14.3)	14.1 (13.8–14.4)	17.1 (16.8–17.3)
Depression	8.9 (8.2–9.5)	15.3 (14.9–15.7)	29.0 (28.6–29.4)	19.0 (18.7–19.3)
Diabetes	40.5 (39.3–41.6)	45.3 (44.8–45.9)	44.8 (44.3–45.2)	22.0 (21.7–22.3)
Heart failure	7.2 (6.6–7.8)	11.2 (10.9–11.6)	10.3 (10.0–10.5)	8.3 (8.1–8.5)
Hyperlipidemia	72.5 (71.5–73.5)	65.1 (64.7–65.6)	69.7 (69.3–70.1)	73.2 (72.9–73.5)
Hypertension	74.3 (73.3–75.4)	87.6 (87.3–87.9)	81.0 (80.7–81.4)	69.2 (68.9–69.5)
Ischemic heart disease	20.3 (19.4–21.3)	18.7 (18.3–19.1)	21.5 (21.2–21.9)	23.5 (23.2–23.8)
Lung cancer	1.7 (1.4–2.0)	0.8 (0.7–0.9)	0.7 (0.6–0.8)	1.8 (1.7–1.9)
Osteoporosis	26.1 (25.1–27.1)	10.9 (10.5–11.2)	19.4 (19.0–19.8)	27.8 (27.5–28.1)
Prostate cancer	—	—	—	––
Stroke	6.7 (6.1–7.3)	7.3 (7.1–7.6)	6.8 (6.5–7.0)	6.8 (6.7–7.0)
**Male sex, no.**	5,636	17,876	26,786	62,686
**Age, no. (%), y**				
50–64	2,258 (40.06)	8,500 (47.55)	11,989 (44.76)	18,815 (30.01)
≥65	3,378 (59.94)	9,376 (52.45)	14,797 (55.24)	43,871 (69.99)
**Poverty level, no. (%)^a^**				
Low	1,354 (24.02)	2,460 (13.76)	2,200 (8.21)	25,212 (40.22)
Medium	2,880 (51.10)	7,616 (42.60)	9,716 (36.27)	29,415 (46.92)
High	1,088 (19.30)	4,438 (24.83)	7,971 (29.76)	6,543 (10.44)
Very high	312 (5.54)	3,360 (18.80)	6,896 (25.74)	1,498 (2.39)
Unknown	2 (0.04)	2 (0.01)	3 (0.01)	18 (0.03)
**Comorbidities, % (95% CI)**				
AMI	7.4 (6.7–8.1)	4.9 (4.6–5.2)	5.7 (5.5–6.0)	6.1 (5.9–6.3)
Arthritis	25.3 (24.2–26.5)	32.8 (32.1–33.5)	30.5 (30.0–31.1)	36.5 (36.2–36.9)
Asthma	8.9 (8.1–9.6)	11.8 (11.4–12.3)	13.5 (13.1–14.0)	9.4 (9.1–9.6)
Atrial fibrillation	20.4 (19.3–21.4)	17.4 (16.8–17.9)	17.8 (17.3–18.3)	28.3 (28.0–28.7)
Breast cancer	0.1 (0.0–0.2)	0.2 (0.1–0.2)	0.1 (0.1–0.1)	0.2 (0.1–0.2)
Chronic kidney disease	24.1 (23.0–25.3)	32.7 (32.0–33.4)	25.9 (25.4–26.4)	15.8 (15.5–16.1)
Colorectal cancer	2.0 (1.7–2.4)	1.8 (1.6–2.0)	1.9 (1.7–2.0)	1.9 (1.8–2.0)
COPD	14.1 (13.2–15.0)	14.1 (13.6–14.6)	13.3 (12.9–13.7)	16.3 (16.0–16.6)
Depression	5.9 (5.3–6.6)	11.5 (11.0–11.9)	16.6 (16.2–17.0)	13.2 (13.0–13.5)
Diabetes	51.0 (49.7–52.3)	48.9 (48.1–49.6)	50.6 (50.0–51.2)	29.7 (29.3–30)
Heart failure	12.2 (11.3–13.0)	16.8 (16.2–17.3)	16.6 (16.1–17.0)	12.8 (12.6–13.1)
Hyperlipidemia	78.9 (77.8–80.0)	66.5 (65.8–67.2)	73.8 (73.3–74.4)	79.0 (78.7–79.3)
Hypertension	82.8 (81.9–83.8)	88.0 (87.5–88.4)	85.8 (85.4–86.2)	78.4 (78.1–78.8)
Ischemic heart disease	43.3 (42.0–44.6)	26.8 (26.2–27.5)	34.3 (33.7–34.8)	43.2 (42.8–43.5)
Lung cancer	2.0 (1.6–2.3)	0.8 (0.6–0.9)	0.7 (0.6–0.8)	1.4 (1.3–1.5)
Osteoporosis	2.9 (2.4–3.3)	1.9 (1.7–2.1)	2.5 (2.3–2.7)	3.7 (3.6–3.9)
Prostate cancer	4.3 (3.8–4.8)	14.3 (13.8–14.8)	8.1 (7.8–8.5)	9.1 (8.9–9.4)
Stroke	8.7 (7.9–9.4)	8.6 (8.2–9.0)	8.2 (7.9–8.5)	7.7 (7.5–7.9)

Abbreviations: — not applicable; AMI, acute myocardial infarction; COPD, chronic obstructive pulmonary disease; ZCTA, Zip Code Tabulation Area.

a Poverty level defined as ZCTA residents living below the federal poverty level: low, <10%; medium, 10% to <20%; high, 20% to <30%; and very high, ≥30%.

The second leading comorbidity profile was hypertension, hyperlipidemia, and diabetes (4%) (Figure). This was the second leading profile across all sex and racial and ethnic groups except White women and men, where hypertension, hyperlipidemia, and arthritis and hypertension, hyperlipidemia, and ischemic heart disease were the second most common profiles, respectively (https://github.com/condes01/covid-shutdown). Prevalence of diabetes among White women (22.0%) and men (29.7%) was substantially lower than the prevalence among the other sex and racial and ethnic groups (range, 40.5%–51.0%). Differences in disease prevalence persisted after controlling for age (https://github.com/condes01/covid-shutdown).

Baseline cardiometabolic risk factors differed significantly by sex and racial and ethnic group (Table 2). Mean SBP and the proportion with high blood pressure was highest among Black women (mean = 134.8 mm Hg, proportion = 33.3%) and men (mean = 134.8 mm Hg, proportion = 33.7%). Across all racial and ethnic groups, women had a higher average LDL than men. Mean HbA1c and the proportion with elevated HbA1c was lowest among White women (mean = 6.0%, proportion = 18.8%) and men (mean = 6.2%, proportion = 26.3%). Obesity was lowest among Asian women (16.5%) and men (15.7%) and highest among Black women (56.6%).

**Table 2:** Cardiometabolic Risk Factors Among New York City Residents Aged 50 Years or Older with Multiple Chronic Conditions Receiving Care at NYC INSIGHT Facilities, by Patient Sex and Race and Ethnicity, 2019

Cardiometabolic risk factor	Asian, mean (SD)		Black, mean (SD)		Hispanic, mean (SD)		White, mean (SD)	
	Female	Male	Female	Male	Female	Male	Female	Male
Systolic blood pressure, mm Hg^a^	128.1 (14.4)	128.7 (14.2)	134.8 (14.7)	134.8 (15.1)	131.8 (14.3)	132.8 (14.6)	128.6 (14.2)	129.2 (13.4)
LDL cholesterol, mg/dL^a^	100.5 (35.3)	85.1 (33.1)	107.4 (35.9)	97.2 (35.7)	104.8 (36.3)	91.0 (34.8)	106.0 (34.8)	89.5 (32.7)
HbA1c, %^a^	6.5 (1.2)	6.7 (1.3)	6.6 (1.5)	6.8 (1.7)	6.6 (1.5)	6.8 (1.7)	6.0 (1.0)	6.2 (1.1)
Weight, lbs^a^	136.5 (28.2)	163.2 (30.1)	185.6 (45.1)	201.2 (46.1)	164.3 (37.3)	186.4 (39.3)	158.9 (39.1)	193.4 (39.4)
High blood pressure, %^a^^,^ b	19.4 (0.5)	19.2 (0.5)	33.3 (0.3)	33.7 (0.4)	25.9 (0.2)	28.6 (0.3)	19.6 (0.1)	19.7 (0.2)
High cholesterol, %^a^^,^ c	42.1 (0.8)	41.3 (1.0)	36.6 (0.3)	31.6 (0.5)	49.5 (0.3)	44.0 (0.4)	36.6 (0.2)	34.8 (0.3)
Elevated HbA1c, %^a^^,^ d	35.1 (0.9)	44.3 (1.0)	36.4 (0.3)	41.3 (0.5)	38.0 (0.3)	44.7 (0.4)	18.8 (0.2)	26.3 (0.3)
Obesity, %^a^^,^ e	16.5 (0.5)	15.7 (0.5)	56.6 (0.3)	40.5 (0.4)	46.5 (0.2)	38.4 (0.3)	34.6 (0.2)	36.3 (0.2)

Abbreviations: BMI, body mass index; HbA1c, hemoglobin A1c; HDL, high-density lipoprotein; LDL, low-density lipoprotein; SD, standard deviation.

a Significant at *P* < .05 using analysis of variance tests for continuous outcomes and χ^2^ tests for categorical outcomes.

b High blood pressure defined as mean annual systolic blood pressure ≥140 mm Hg.

c High cholesterol defined as mean annual LDL cholesterol ≥160 mg/dL; HDL <40 mg/dL male or <50 mg/dL female; total cholesterol ≥240 mg/dL; or triglyceride ≥200 mg/dL.

d Elevated HbA1c defined as mean annual HbA1c ≥6.5%.

e Obesity defined as mean BMI ≥30 kg/m^2^.

## Discussion

We assessed burden and comorbidity profiles of NYC older adult patients with MCCs and observed potentially important disparities in MCC burden and cardiometabolic risk factors across the intersection of race and ethnicity and sex, with increased burden of hypertension among Black men and women and decreased burden of diabetes among White men and women. Results were generally consistent with patterns observed in a study of comparably aged English adults that examined similar risk factors by the intersection of gender, ethnicity, education, and income (7). The most common comorbidity profile was the co-occurrence of hypertension and hyperlipidemia, and Asian men and women had lowest prevalence of obesity yet high prevalence of diabetes, consistent with prior research (5,8).

Within all racial and ethnic categories, LDL was higher among women than men, yet a higher proportion of men had a hyperlipidemia diagnosis. These findings could be partially attributed to the use of lipid-lowering medications, where men and White patients consistently report higher usage than women or patients of racial and ethnic minorities (9). Additionally, research suggests that there may be biological differences in the pharmacological effects of lipid-lowering medications by sex (10). These observed differences in cholesterol levels by sex and racial and ethnic groups could reflect a combination of structural discrimination and biological factors that influence statin efficacy.

As with any EHR-based study, key limitations need to be considered. Disease status may be misclassified, particularly among patients who are seen less frequently, as documentation of ICD-10 codes may be influenced by billing practices, and care from institutions external to INSIGHT will not be represented in these data. INSIGHT includes some academic medical centers that disproportionately serve privately insured patients, so our findings may not be generalizable and observed disparities may be underestimated relative to the overall NYC population (11). Additionally, a substantial proportion of patients had an unknown race and ethnicity, and the race and ethnicity distribution of these patients may have been systematically different from those with a recorded race and ethnicity.

Despite these limitations, the large size and diversity of these data provided an opportunity to examine NYC older adults with MCCs across the intersection of race and ethnicity and sex. Findings suggest the existence of significant differences in comorbidity burden, which are associated with meaningfully increased risk of cardiovascular events (7). These differences most likely reflect a combination of biological factors and preventable disparities driven by underlying systems of power and discrimination (12). Future research should explore disparities in continuity and quality of care in the management of older adults with MCCs.
