# Erratum, Vol. 21, August 1 Release

**DOI:** 10.5888/pcd22.230444e

**Published:** 2025-07-03

In the article, “Descriptive Epidemiology of New York City Older Adult Patients With Multiple Chronic Conditions,” some of the information reported in the Results section was not correct and did not match the supplemental information provided (https://github.com/condes01/covid-shutdown).

The text of the article incorrectly reported that the second leading comorbidity profile for White men was hypertension, hyperlipidemia, and arthritis, and that the second leading comorbidity profile for White women was hypertension, hyperlipidemia, and ischemic heart disease. However, the second leading comorbidity profile for White men was hypertension, hyperlipidemia, and ischemic heart disease, and the second leading comorbidity profile for White women was hypertension, hyperlipidemia, and arthritis. This information was correctly reported in the supplemental information.

The correction was made to our website on June 24, 2025, and appears online at https://www.cdc.gov/pcd/issues/2024/23_0444.htm. We regret any confusion or inconvenience this error may have caused.

